# Body composition and determinant factors among mother–child pairs (6–8 months) in rural areas of Senegal

**DOI:** 10.1111/mcn.13174

**Published:** 2021-03-14

**Authors:** Abdou Badiane, Adama Diouf, Papa M. D. D. Sylla, Ndeye S. Cissé, Nicole Idohou‐Dossou, Michèle Dramaix, Salimata Wade, Philippe Donnen

**Affiliations:** ^1^ Laboratoire de Recherche en Nutrition et Alimentation Humaine (LARNAH), Département de Biologie Animale, Faculté des Sciences et Techniques Université Cheikh Anta Diop (UCAD) Dakar Senegal; ^2^ Laboratoire de Botanique et Biodiversité, Département de Biologie Végétale, Faculté des Sciences et Techniques Université Cheikh Anta Diop (UCAD) Dakar Senegal; ^3^ Laboratoire de Recherche sur les Transformations Economiques et Sociales, Institut Fondamental d'Afrique Noire Université Cheikh Anta Diop (UCAD) Dakar Senegal; ^4^ Centre de Recherche Politiques et systèmes de Santé ‐ Santé Internationale, Ecole de Santé Publique Université Libre de Bruxelles Bruxelles Belgium

**Keywords:** body composition, food consumption, infant and child nutrition, low‐income countries, maternal nutrition, socio‐economic factors

## Abstract

This cross‐sectional study was conducted to determine the association between selected characteristics and body composition of mothers and children in early life. This study included 213 mother–child pairs 6 to 8 months involving in the cohort study of the Research and Development Project conducted in Kaffrine district. The main outcomes were fat‐free mass (FFM) and body fat (BF), measured using deuterium dilution method and anthropometry. Independent variables were sociodemographic, dietary diversity and health characteristics. Descriptive, correlation, bivariate and multiple regression analyses were conducted. According to body mass index (BMI), 23% of mothers were underweight, 12% were overweight/obese and 11% had excess BF. Four per cent of children were below −2 weight‐for‐length *z*‐score (WLZ), 10% were below −2 length‐for‐age *z*‐score (LAZ) and 40% had excess BF. Maternal FFM was positively correlated with child FFM (*r* = 0.25, *P* = 0.002). Similarly, mothers' BMI, FFM and BF were significantly and positively correlated with children's LAZ. Stepwise regression showed an increased association between minimum dietary diversity (MDD) and WLZ score, FFM and BF of children. Among mothers, being employee and doing reproductive health care were determinants of higher BMI, FFM and BF. This study found a strong association between maternal and child body composition in early life. Adequate diet is the main determinant of children nutritional status. Among the mothers, having a job and doing primary health care seem to be beneficial for the nutritional status. Improvement of women's empowerment, quality of health care and dietary diversity could have a positive impact on maternal and child nutrition.

**Key messages**

- The first 1000 days of life (antenatal + first 2 years) and even preconception period are critical period able to influence later body composition and have lifelong consequences in terms of overall health, physical activity patterns and work productivity.
- Studies on body composition component, using method of stable isotope dilution, are lacking and challenging among mothers and children in early life.
- Maternal body composition has an influence on offspring nutritional status.
- A good food diversity should improve nutritional status of children.
- The determination of body composition and its related determinant factors is important among mother–child pairs in early life.

## 1 BACKGROUND

From conception to old age, body composition is constantly changing (Forbes, 1987), at atomic, molecular, cellular, tissue and whole‐body level (Wang et al., 1992). Many studies have identified the first 4–9 months of life as a critical period able to influence later body composition (Black et al., 2013; Ejlerskov et al., 2015; Eriksen et al., 2013). Body composition patterns in childhood can have lifelong consequences in terms of overall health, physical activity patterns and work productivity (Ejlerskov et al., 2015; Wells et al., 2009; Zemel & Bruckbauer, 2013). For example, among school‐aged boys in Colombia, the group of poorly nourished boys had less spontaneous physical activity than adequately nourished boys (Spurr & Reina, 1988). Similarly, among children with sickle cell disease who have reduced fat‐free mass (FFM) and fat mass (FM), total energy used for physical activity was lower than in healthy controls (Barden et al., 2000). Among the mothers, the prenatal and postnatal periods coincide with an important change in the body composition and can often lead to wasting or overweight/obesity. Data are emerging to suggest impact of maternal body composition on offspring nutritional status. New evidence reinforces the importance of maternal nutritional status, both for the health of the mother and for ensuring healthy child growth (Negash et al., 2015).

There is still much to be learned about the relationship between body composition and diet. The essential nutrients required for normal growth and cell functioning assure normal body composition. Clearly, when severe nutrient deficiencies exist, body composition is altered (Wells et al., 2009). The association between diet patterns and body composition is even less well defined. For example, Zemel and Bruckbauer (2013) showed that vegans, who exclude all animal products from their diet, are leaner still than both vegetarians and omnivores, and vegetarians have less body fat (BF) than omnivores. A positive correlation between good nutritional status and high food diversity has been reported in breastfed children (Butte et al., 2000; Tang & Krebs, 2014). Marinangeli and Jones (2012) showed that legume consumption, especially pulse grains, had beneficial effects on the fat deposition and on the prevention and management of obesity‐related metabolic disorders, because of their low‐energy density and high nutritional properties. Likewise, Wells et al. (2009) found that the lack of some micronutrients in the diet, particularly zinc and iron, leads to the reduction of lean mass and the accumulation of BF in Gambian's children. Excess adiposity is associated with high energy intake, consumption of energy‐dense, highly processed foods, low fruit and vegetable intake and consumption of sugared beverages (Gillis et al., 2002). Both lower BF and improved bone mineral accrual are associated with diets rich in dark green and deep yellow vegetables and low in fried foods (Wosje et al., 2010). Other key findings like lifestyle, gender, age and socio‐economic characteristics were also found as determinant factors of maternal and child nutritional and body composition status, but results were mitigated (Abubo et al., 2008; Negash et al., 2015).

A two‐component assessment of body composition as obtained by deuterium dilution includes the amount of FFM and FM (Wells & Fewtrell, 2006). Two other indices, the FFM index (FFMI; fat‐free mass/height^2^) and FM index (FMI; fat mass/height^2^), had been proposed to be more predictive of mortality and health risk (Van Itallie et al., 1990). In Senegal, data assessing the body composition component using stable isotope dilution method in early infancy are lacking and challenging. All programmes assessing maternal and child nutritional status were based on anthropometry, and therefore, the nature of their impact on body masses is not clear. A limited number of studies have been carried out among children in the age group 8–11 years and adult living with HIV (Diouf et al., 2016, 2018). It is important to understand these changes in mothers and their offspring at all levels to be able to interpret body composition measurements correctly. Based on these facts, the present cross‐sectional study aimed to determine the body composition using deuterium dilution method and its related determinant factors in mother–child pairs in Kaffrine district, middle area of Senegal. We hypothesized that maternal and child body composition are mutually correlated and influenced by many of their characteristics. This study is part of the Project Research and Development (PRD) set up in Kaffrine district, a cohort study of a nutrition‐sensitive agriculture intervention conducted from January 2017 to October 2018. This paper aimed to present the baseline status of mother–child pairs involved in this study.

## 2 METHODS

### 2.1 Study area and design

‘This study use the baseline data selected of a larger cohort study conducted in rural area of Senegal in order to assess the impact of nutrition‐sensitive agriculture intervention on the prevention of malnutrition during the first 1000 days of life’ (Badiane et al., 2018).

This baseline survey was conducted between January and May 2017 in three villages (Sagna, Malem Hodar and Kathiotte) of Kaffrine district, the central groundnut basin of Senegal. In brief, all mother–child pairs aged from 6 to 8 months who had been residents of the study area for more than 6 months were invited to participate in the study at baseline. To select mother–child pairs, the recruitment took place gradually from January to May 2017. Due to the long recruitment period (January to May), the selection was finally stopped to 213 mother–child pairs aged 6–8 months randomly chosen in the three villages. However, data were not presented by allocated village.

### 2.2 Anthropometric measurements

Anthropometric measurements were performed using standardized procedures. Body weight was measured to the nearest 0.1 kg using electronic scales (SECA 869, GmbH & Co, Hamburg, Germany). The mothers were weighed with a light loincloth, and the children were measured without clothes. Height of mothers and length of children were measured to the nearest 0.1 cm with a Seca board (SECA 217, California, USA). From anthropometric measures, length‐for‐age *z*‐scores (LAZ) and weight‐for‐length *z*‐scores (WLZ) were calculated, and children were classified as stunted (LAZ < −2) or wasted (WLZ < −2) using the World Health Organization (WHO) child growth standard reference (WHO, 2006). Among the mothers, body mass index (BMI; kg/m^2^) was used to categorize them as underweight (<18.5), normal weight (18.5 to 24.9) and overweight/obese (≥25.0) (OMS, 1996).

### 2.3 Body composition measurement

Briefly, baseline saliva samples were collected (predose) for both mothers (after 8 h of fasting) and infants (1 h after the last feeding) by rotating a cotton‐wool ball in the buccal cavity of the mouth until well soaked. Saliva was collected into a clean sterile and dry tube (Thermo Fisher Scientific, Denmark) using a 20‐ml disposable syringe (Norm‐ject, Henke‐Sass Wolf, Tuttlingen, Germany). After predose samples, deuterium oxide‐labelled water (99.8% purity; Cambridge Isotope Laboratories Inc., Andover, USA) accurately weighed (0.001‐g precision) was orally administered to the children according to their body weight (0.5 g of deuterium oxide/kg) and 30‐g D_2_O followed by 50 ml of local tap water to the mothers. The dose was given to mothers using a polystyrene pot and straw, whereas to children, it was given directly using a 10‐ml syringe. After the dose, mothers were asked not to give food/drink or breastfeeding to their children for at least 30 min before receiving the deuterium‐labelled water and to void their bladders before dosing. Two saliva samples (postdose) were collected from both mothers (3 and 4 h) and children (2 h 30 min and 3 h) using the method described above. All saliva samples were put in Ziploc bags and stored at 4°C until their arrival to the laboratory for storage at −20°C until measurement. Deuterium enrichment of the saliva samples was measured using a Fourier Transform Infrared Spectrometer (FTIR IR‐Affinity, Shimadzu, Nakagyo‐Ku Kyoto, Japan) in accordance with IAEA protocols (IAEA, 2010). Total body water (TBW) was calculated from D_2_O dilution space using a correction factor of ~4% (Forbes, 1987). Fat‐free mass (FFM) was estimated using established age and sex‐specific constants for the hydration of FFM, and BF as well as per cent body fat (%BF) calculated as described elsewhere (IAEA, 2010; Lohman, 1986). These constants used for body composition calculations are valid for this population, as well as for the age groups used. Quality control procedures with stringent criteria were described in detail elsewhere (IAEA, 2010). Excess BF was defined by %BF ≥ 35% in mothers (De Lorenzo et al., 2003) and in children according to criteria defined by William et al. (1992). To assess child's adiposity, FFM index (FFMI = FFM/length^2^) and BF index (BFI = BF/length^2^) proposed by Van Itallie et al. (1990) as an analogy of BMI were used and expressed in common units of kg/m^2^.

### 2.4 Questionnaire data collection

A pretested and structured questionnaire was used to assess sociodemographic characteristics, food consumption, health care and morbidity of the mother–child pairs. The questionnaire was developed in French using process validation and then translated into the local language.

Data on food consumption were measured using the 24‐h dietary recall method in one single day per participant by trained investigators. Among the mothers, minimum dietary diversity (MDD) was defined by proportion of mothers who consumed at least five out of the 10 defined food groups: (1) grains, white roots and tubers, and plantains; (2) pulses (beans, peas and lentils); (3) nuts and seeds; (4) dairy; (5) meat, poultry and fish; (6) eggs; (7) dark green leafy vegetables; (8) other vitamin A‐rich fruits and vegetables; (9) other vegetables; and (10) other fruits (FAO, 2016). Among the children, optimal breastfeeding and complementary feeding data were collected using standardized indicators of infant and young child feeding (WHO, 2008). The MDD was defined as the proportion of children who have consumed at least four out of the seven defined food groups the previous 24 h: (1) staples (grains, roots and tubers), (2) legumes and nuts, (3) dairy products (milk, yogurt and cheese), (4) fresh foods (meat, fish, poultry and liver/organ meats), (5) eggs, (6) vitamin A‐rich fruits and vegetables and (7) other fruits and vegetables. Minimum meal frequency was defined as the proportion of children who received during the previous 24 h two meals for breastfed children and four meals for nonbreastfed children.

### 2.5 Data analysis

The data were entered, checked for missing values and outliers and analysed with STATA, Version 14.0. The weights and lengths of the children were converted to *z*‐scores with WHO Anthro Software, Version 1.0.4. Because of the difference in body composition, results were stratified by sex in children and according to mothers' age group (<20 and ≥20 years). The age range of the mothers was based on the definition of WHO that defines ‘adolescents’ as individuals in the 10–19 years age group; therefore, mothers were ranged either in <20 years group or in ≥20 years group. Independent sample *t* test, Pearson's *χ* ^2^ test and Fisher's exact test were used to compare data between mothers' age group or boys and girls.

Pearson's correlation coefficient was used to examine the relationship between body composition data and selected anthropometric variables. The regression line with its 95% confidence interval (95% CI) was represented in the figure. To identify the determinants of mother and child body composition, only variables that were associated at *P* < 0.20 in the bivariate analyses were entered into backward stepwise multivariable linear regression models. All of the conditions in the multiple regression analysis were checked (i.e., assumptions about the linearity of the model, normal distribution of residuals, homoscedasticity and absence of outliers). *P* values less than 0.05 were considered statistically significant.

### 2.6 Ethical considerations

The study protocol was approved by the national ethical committee for research of the Ministry of Health in Senegal under the number 178/MSAS/DPRS/CNERS. Women and children were eligible if informed written consent was provided by the heads of household and women or children caregivers.

## 3 RESULTS

### 3.1 Characteristics of the study population

Characteristics of the participants are presented in Table 1. Of the 213 mother–child pairs recruited, three had missed saliva sample collection due to a lack of time to devote to the study. In addition, body composition data from two children were deleted in the dataset because they did not fit to the quality control grounds of TBW measurement. Finally, statistical analysis data were limited to the 208 completed pairs.

**TABLE 1:** Sociodemographic, health and morbidity characteristics of the mother–child pairs

	% or M ± SD^a^
**Mother characteristics (*n* = 208)**	
Age (years)	25 ± 6
<20	21.6
≥20	78.4
Ethnicity	
Wolof	94.2
Other	5.8
Maternal occupation	
Unemployed (housewife, student)	80.3
Employed (farmer, seller, dressmaker, …)	19.7
Education	
No formal education	24.5
Formal education (primary/secondary)	15.6
Koranic	59.6
Reproductive health care before the last pregnancy (yes)	46.2
Parity	
Primiparous	24.0
Multiparous	76.0
Number of children <5 years alive	
1	24.0
2	11.1
3–4	64.9
Iron/folate postpartum supplementation (yes)	11.1
Antenatal care (yes)	92.8
Postnatal care (yes)	91.8
**Children characteristics (*n* = 208)**	
Age (months)	6.8 ± 0.8
Sex (female)	50.1
VAS (last 6 months) (yes)	51.7
Deworming tablet (last 6 months) (yes)	21.4
Complete immunization^b^	96.6
Morbidity (last 2 weeks)	
Fever	22.7
Diarrhoea	24.6
Acute respiratory infection	16.4
BW (g), (*n* 137)	3146 ± 517
Low birth weight	6.6

Abbreviations: BW, birth weight; VAS, vitamin A supplementation.

^a^ Values are expressed in percentage for nominal data, mean (SD) for data normally distributed or median [interquartile] for data not normally distributed.

^b^ Both hepatitis B, BCG, polio, pentavalent and pneumococcal vaccines.

Results showed that the majority of women were at least 20 years old (78.4%), belong to the Wolof ethnic group (94.2%), were homemakers (80.3%), multiparous (76.0%) and had followed the antenatal care during their last pregnancy. Less than 50% of them had used reproductive health care before the last pregnancy, and only 11% had received iron/folate postpartum supplementation after given birth. Among the children, the gender was quite balanced. Half of the children had received a high dose of vitamin A in the last 6 months, and the majority received their first vaccines. Regarding morbidity data during the last 2 weeks, 22.7% of the children had fever, one quarter suffered from diarrhoea and 16.4% presented cough.

### 3.2 Food consumption of the mother–child pairs

**FIGURE 1:**
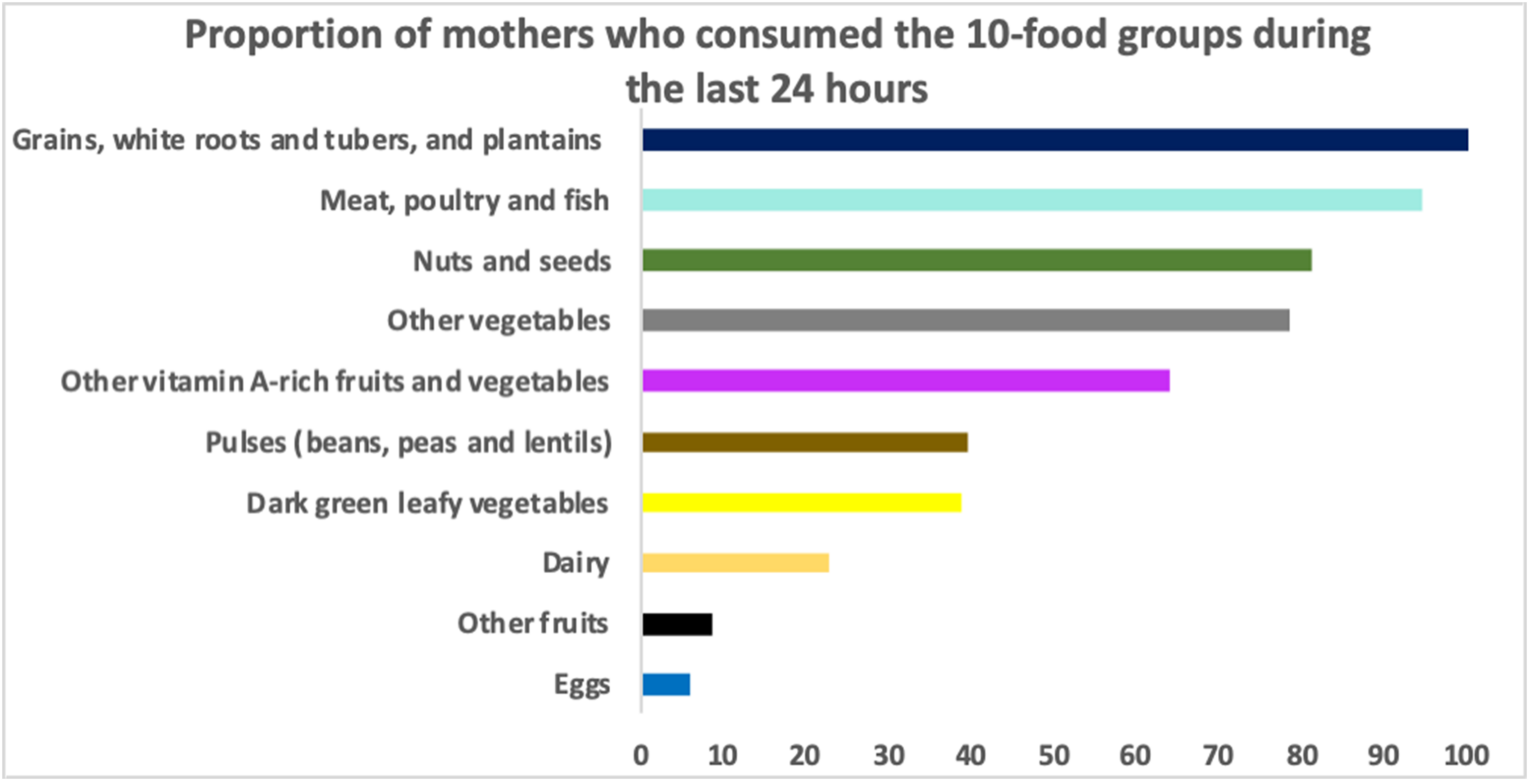
Proportion of women who consumed the 10‐food groups in the last 24 h (*n* = 208)

Among the mothers, the mean (±SD) dietary diversity score was 5.2 ± 1.5, and 67.3% of them had reached MDD. The 24‐h dietary recall (Figure 1) revealed that the mothers' diet was characterized by the consumption of the five following food groups: starchy foods (rice and millet), meat, poultry and fish (fresh or dried smoked fish), nuts and seeds (peanut butter of flour), other vitamin A‐rich fruits and vegetables (carrot) and other vegetables (tomato and onion). In addition to these food groups, more than 50% had consumed vegetable oil or palm oil. Pulses (cowpeas) and dark green leafy vegetables (moringa leaves) food groups were consumed by less than 40% of the mothers. The consumption of dairy products and eggs were low, only by 23.1% and 5.8% of mothers, respectively.

**FIGURE 2:**
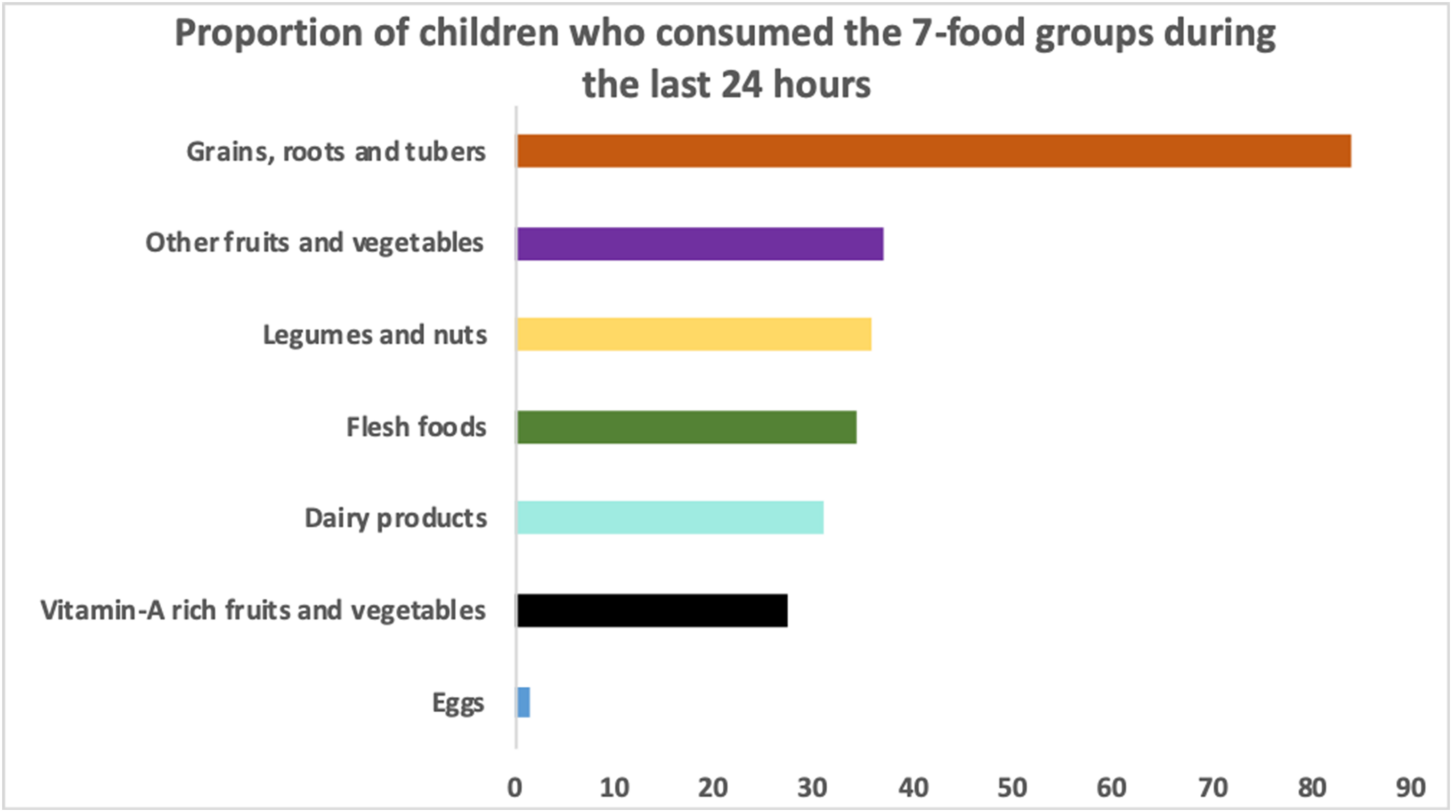
Proportion of children who consumed the seven‐food groups in the last 24 h (*n* = 208)

Almost all the children continued to be breastfed after 6 months. Most of them started receiving complementary food. Forty per cent received minimum meal frequency, but only 15.9% had achieved MDD. The 24‐h dietary (Figure 2) recall revealed that more than 80% of the children had consumed starchy foods essentially rice or millet porridge. One third had consumed legumes and nuts (peanut butter), other fruits and vegetables (tomato and onion), flesh foods (fresh or dried smoked fish) and dairy products (powdered milk). Consumption of vitamin A‐rich fruits and vegetables food groups was low (18.8%). Only two children (1%) had consumed eggs.

### 3.3 Anthropometry and body composition of the mother–child pairs

Mothers' mean BMI was 21.0 ± 3.3 kg/m^2^, and according to WHO classification, 23.0% and 12.0% of mothers were underweight and overweight/obese, respectively (Table 2). Those under 20 years tended to have a higher proportion of underweight and a lower proportion of overweight/obese compared with their older peers. Data from body composition showed that BF and %BF were significantly higher in mothers aged 20 years and above compared with those of <20 years. Proportion of mothers with excess BF (%BF > 35) was also significantly higher in among those aged ≥20 years compared with their younger peers. In contrast, mean FFM was comparable between the two age groups.

**TABLE 2:** Anthropometry and body composition of the mother–child pairs

Mothers	All (*n* = 208)	<20 years (*n* = 46)	≥20 years (*n* = 167)	*P* value^a^
Anthropometry^b^				
BMI (kg/m^2^), M ± SD	21.0 ± 3.2	20.2 ± 2.5	21.3 ± 3.4	*0.057*
Underweight, %	23.0	33.33	21.5	*0.083*
Normal weight, %	62.2	64.4	63.9	
Overweight/obese, %	12.0	4.0	14.1	
Body composition				
FFM (kg), M ± SD	40.9 ± 4.9	40.4 ± 4.4	41.1 ± 5.0	*0.454*
BF (kg), M ± SD	16.1 ± 6.4	13.9 ± 5.0	16.8 ± 6.9	***0.011***
%BF, M ± SD	27.4 ± 6.9	25.2 ± 6.2	28.1 ± 6.9	***0.012***
Excess body fat, %	10.6	2.2	12.9	***0.027***

Children	All (*n* = 208)	Boys (*n* = 102)	Girls (*n* = 106)	*P* value^a^
Anthropometry^b^				
WLZ, M ± SD	−0.3 ± 1.0	−0.5 ± 1.0	−0.2 ± 1.0	**0.055**
Wasting, %	3.8	5.9	1.9	*0.128*
LAZ, M ± SD	−0.5 ± 1.1	−0.6 ± 1.1	−0.4 ± 1.1	*0.133*
Stunting, %	9.6	9.8	9.4	*0.928*
Body composition				
FFM (kg), M ± SD	5.5 ± 0.7	5.7 ± 0.7	5.3 ± 0.7	***<0.001***
FFM index (kg/m^2^), M ± SD	12.2 ± 1.1	12.5 ± 1.0	12.0 ± 1.2	***0.005***
BF (kg), M ± SD	1.9 ± 0.6	1.9 ± 0.6	1.9 ± 0.7	*0.751*
BF index (kg/m^2^), M ± SD	4.3 ± 1.3	4.2 ± 1.2	4.4 ± 1.4	*0.160*
%BF, M ± SD	25.7 ± 6.5	24.8 ± 5.8	26.6 ± 7.0	***0.049***
Excess body fat, %	40.4	48.0	33.0	***0.027***

Abbreviations: BF, body fat; BMI, body mass index; FFM, fat‐free mass; LAZ, length‐for‐age *z*‐score; WLZ, weight‐for‐length *z*‐score.

^a^ *P* value obtained using independent sample *t* test, or *χ* ^2^ or Fisher's exact *t* test between boys and girls or between mothers <20 years and mothers ≥20 years.

^b^ Underweight: BMI < 18.5 kg/m^2^; normal weight: 18.5 ≥ BMI < 25 kg/m^2^; overweight/obese: BMI ≥ 25 kg/m^2^; wasting: WLZ > −2; stunting: LAZ < −2; excess body fat: mother = %BF > 35%, boy = %BF > 25%, girl = %BF > 30%.

Among the children, 3.8% and 10% were wasted and stunted, respectively (Table 2). Sex difference was observed in mean WLZ, which was higher in girls (*P* < 0.05), but the prevalence of wasting and stunting was comparable. Body composition data showed that boys had higher FFM and FFMI and lower %BF than girls. In addition, excess BF was also significantly higher in boys than in girls (*P* = 0.027).

**FIGURE 3:**
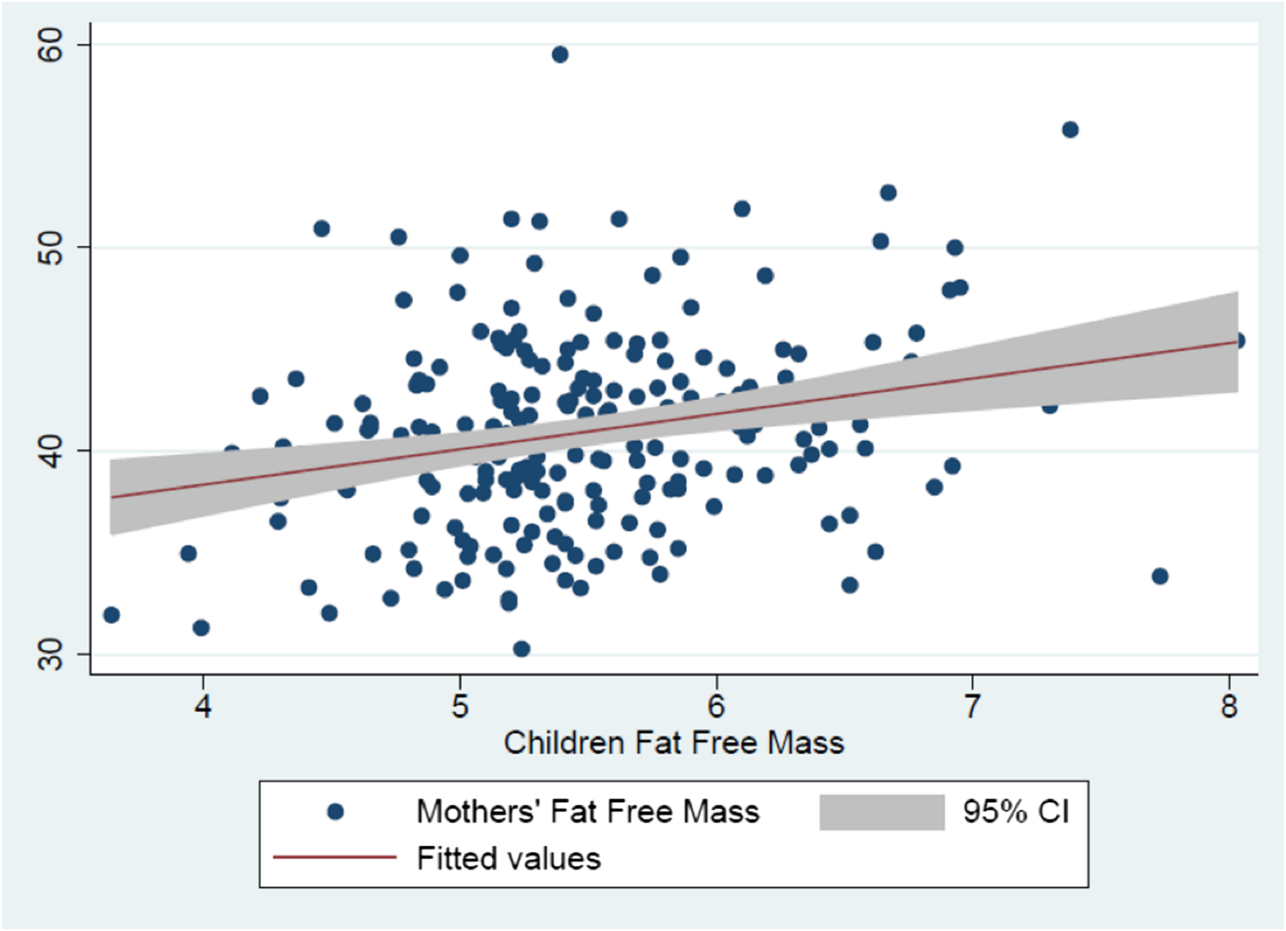
Regression of mothers' fat‐free mass and regression of children's fat‐free mass correlation between maternal and child's fat‐free mass (*n* = 208). CI, confidence interval

The correlation between anthropometry and body composition variables was tested. In Table 3, Pearson's correlation coefficient was positive between children's FFM and mothers' FFM (*r* = 0.25; *P* = 0.0002). The trend in Figure 3 indicates a linear relationship for these variables within the limits of observation. LAZ of children was significantly and positively correlated with BMI and all body composition parameters of mothers. However, no relationship was found between WLZ of children and any anthropometric or body composition variables of mothers.

**TABLE 3:** Correlation coefficients between anthropometry and body composition of mother–child pairs (*n* = 208)

	WLZ	LAZ	Child FFM	Child BF	Child %BF	Mother BMI	Mother FFM	Mother BF
WLZ								
LAZ	0.23**							
Child FFM	0.41**	0.54**						
Child BF	0.68**	0.41**	0.13					
Child %BF	0.49**	0.220**	−0.25**	0.92**				
Mother BMI	0.06	0.20*	0.13	0.02	−0.02			
Mother FFM	0.08	0.25**	0.25**	0.03	−0.07	0.59**		
Mother BF	0.02	0.24**	0.09	0.06	0.04	0.89**	0.40**	
Mother %BF	0.02	0.21**	0.03	0.07	0.07	0.75**	0.10	0.94**

Abbreviations: BF, body fat; BMI, body mass index; FFM, fat‐free mass; LAZ, length‐for‐age *z*‐score; WLZ, weight‐for‐length *z*‐score.

* *P* < 0.05.

** *P* < 0.01.

### 3.4 Predictors of mother–child pairs nutritional status

All sociodemographic, health and dietary diversity variables that were significant at *P* < 0.20 were entered into stepwise linear regression analyses. Among the mothers, the education level and MDD were not retained as independent variables in the stepwise selection. Maternal age and parity were non‐significant in all final models; reproductive health cares were not significantly associated with FFM. Results from stepwise selection showed that being employed was significantly associated with high maternal BMI whereas lack of reproductive health cares were associated with low BMI of mothers (Table 4). High BF and %BF were each associated with being employed, doing reproductive health care and antenatal care attendance. High FFM of mothers was associated with maternal occupation (employed).

**TABLE 4:** Determinants of anthropometry and body composition variables among the mothers

	*β* coefficient	95% CI	*P* value
**BMI (*R*** ^**2**^ **= 12.9%, *n* = 208)**			
Reproductive health care			
Yes	0		
No	−1.17	−2.01, −0.33	*0.004*
Maternal occupation			
Unemployed (housewife, student)	0		
Employed (farmer, seller, …)	1.88	0.83, 2.93	*0.001*
Antenatal care			
Yes	0		
No	−2.72	−4.34, −1.09	*0.001*
**FFM (*R*** ^**2**^ **= 3.0%, *n* = 208)**			
Maternal occupation			
Unemployed (housewife, student)	0		
Employed (farmer, seller, …)	2.14	0.48, 3.80	*0.012*
**BF (*R*** ^**2**^ **= 14.0%, *n* = 208)**			
Reproductive health care			
Yes	0		
No	−2.99	−4.65, −1.34	*<0.001*
Maternal occupation			
Unemployed (housewife, student)	0		
Employed (farmer, seller, …)	3.30	1.23, 5.36	*0.002*
Antenatal care			
Yes	0		
No	−5.45	−8.63, −2.27	*0.001*
**%BF (*R*** ^**2**^ **= 9.9%, *n* = 208)**			
Reproductive health care			
Yes	0		
No	−2.89	−4.69, −1.08	*0.002*
Maternal occupation			
Unemployed (housewife, student)	0		
Employed (farmer, seller, …)	2.69	0.44, 4.95	*0.019*
Antenatal care			
Yes	0		
No	−4.85	−8.33, −1.38	*0.006*

*Note*: The independent variables not retained for stepwise selection were education level and minimum dietary diversity. Maternal age and parity were non‐significant in all final models; reproductive health care and antenatal care were not significantly associated with FFM.

Abbreviations: BF, body fat; BMI, body mass index; CI, confidence interval; FFM, fat‐free mass.

Among the children, the independent variable not retained for stepwise selection was child's illness. The final models of regression have retained child's sex and MDD as significant determinants of anthropometry and child body composition. In Table 5, data showed that being girl was significantly associated with high WLZ score whereas being boy was positively associated with increase of FFM and FFMI. MDD in children was significantly associated with high WLZ, BF and %BF but not with variation of FFM, FFMI and LAZ.

**TABLE 5:** Determinants of anthropometry and body composition variables among the children

	*β* coefficient	95% CI	*P* value^a^
**WLZ (*R*** ^**2**^ **= 5.6%, *n* = 208)**			
Sex			
Male	0		
Female	0.29	0.01, 0.56	*0.040*
Minimum dietary diversity			
No	0		
Yes	0.52	0.17, 0.90	*0.006*
**FFM (*R*** ^**2**^ **= 10.1%, *n* = 208)**			
Sex			
Male	0		
Female	−0.45	−0.63, −0.26	*<0.001*
**FMMI (*R*** ^**2**^ **= 3.7%, *n* = 208)**			
Sex			
Male	0		
Female	−0.42	−0.73, −0.12	*0.006*
**BF (*R*** ^**2**^ **= 3.4%, *n* = 208)**			
Minimum dietary diversity			
No	0		
Yes	0.33	0.09, 0.57	*0.008*
**%BF (*R*** ^**2**^ **= 3.8%, *n* = 208)**			
Minimum dietary diversity			
No	0		
Yes	2.50	0.10, 4.90	*0.041*
Sex			
Male	0		
Female	1.75	−0.01, 3.51	*0.051*

*Note*: The independent variable not retained for stepwise selection was child's illness. Minimum dietary diversity was not significantly associated with FFM, FFMI and BF.

Abbreviations: BF, body fat; CI, confidence interval; FFM, fat‐free mass; FFMI, fat‐free mass index; WLZ, weight‐for‐length *z*‐score.

^a^ *P* obtained using multiple linear regression analysis with stepwise model.

## 4 DISCUSSION

This baseline survey was part of an impact evaluation of an agriculture sensitive nutrition intervention undertaken in the rural central groundnut basin of Senegal. The results of this cross‐sectional study showed high prevalence of undernutrition and illness (fever, diarrhoea and cough) in early life (6–8 months) as previously reported by the national demographic and health survey (DHS), while the standard health care packages were still delivered by local medical staffs. Indeed, the last DHS has found a proportion of 7.8% of wasting and 7.9% stunting among the infants 6–8 months (ANSD, 2017). The study showed also a high proportion of undernutrition among lactating women. These findings might be explained by the fact that lactating mothers are at an increased risk of undernutrition compared with their reproductive same‐aged counterparts due to physiological changes during lactation (Mardani et al., 2020) that lead to increased energy and nutrient requirements (Desalegn et al., 2018).

In this study, body composition was measured simultaneously in mother–child pairs for the first time in Africa using deuterium dilution technique as reference method. The results showed a positive and significant association between maternal body composition parameters and LAZ of children and between mothers' BMI and LAZ. These findings were consistent with other epidemiological studies that demonstrated the influence of maternal nutritional status on offspring linear growth (Addo et al., 2013; Lander et al., 2012; Negash et al., 2015; Vir, 2016). According to Vir (2016), maternal undernutrition is estimated to be responsible for about 20% of childhood stunting and can greatly affect the outcome of pregnancy and child health. A study conducted by Silveira et al. (2010) had noted that low height of mothers was associated with child stunting, both in mothers with weight deficits and in those with overweight/obesity. Other authors observed that, in developing countries, children with chronic malnutrition and obese mothers cohabit (Correia et al., 2014; Jehn & Brewis, 2009; Martins et al., 2007; Negash et al., 2015). Our study found also a positive and significant correlation between maternal FFM and that of their children. Indeed, maternal nutritional status is associated with birth and postpartum outcomes such as birthweight, infant morbidity, mortality and body composition (Kamal et al., 2015). This further risks the intergenerational cycle of undernutrition (Christian & Smith, 2018) particularly in situations of social, economic and gender inequities (Perez‐Escamilla et al., 2018), which are highly prevalent in developing countries. Maternal body composition has been shown to affect the secretion of nutrients in breast milk leading to the increased risk of child nutritional status and a long‐term impact on the child's body composition (Haileslassie et al., 2013; Ukegbu, 2014). All of these results indicated that strategies to improve maternal nutrition could have a significant role in the increase of childhood nutrition status and particularly the body composition.

Our results revealed that nutritional status of the children as assessed by body composition and anthropometry indicators was positively and significantly associated with a good dietary diversity. These findings are inconsistently linked to previous studies conducted in children 6–23 months old living in many developing countries (Arimond & Ruel, 2004; Bork et al., 2012; Ruel & Menon, 2002). In Senegal, Bork et al. have showed that 24‐h dietary diversity and food variety were both strongly and positively associated with LAZ at 6–12 months (Bork et al., 2012). The authors concluded that the strong association between LAZ and these food indicators during infancy may be partly due to greater appetite for and interest in nonbreast milk foods among infants. Other study about link between diet quality and risk of stunting in low‐ and middle‐income countries has showed that children aged 6–23 months who did not consume animal source food (ASF) had 1.4 higher odds of being stunted than those who consume egg, meat and dairy (Krasevec et al., 2017).

Among the mothers, we found no association between dietary diversity and their nutritional status. These findings were different with most of causality studies, probably due in our study to the mothers' diet, which consisted in high energy‐dense foods and low‐quality diets characterized by imbalance in intake of protein and micronutrients (George et al., 2014; IOM, 2005; WHO, 2004). Studies from Bangladesh and Mozambique, where dietary patterns are heavily dominated by starchy staples, are in concordance with our study (Arimond & Ruel, 2004). In other part, the absence of association could be explained by the limits of the 24‐h dietary recall method and the fact that we used only one 24‐h recall day instead of 2 or 3 days to be more valid but also to the lack of information on the amounts of food consumed. Other determinants of food dietary such as food insecurity, female illiteracy, poverty and lack of empowerment of women could also explain the lack of association between dietary diversity and maternal nutritional status. The production and consumption of micronutrient rich foods by nutrition sensitive agricultural interventions can help to improve maternal and child nutrition in rural area (Ruel et al., 2013).

In the present study, reproductive health care, occupation and antenatal care emerged as the main determinants of anthropometry and body composition of mothers. All of these factors lead to women having a higher status in the communities, hence having more control over household resources, more access to information and health services, good mental health and higher self‐esteem (Alemayehu et al., 2018; Smith et al., 2003). In this study, having an employment (farmer, seller and dressmaker) was a strong and positive predictor of the maternal nutritional status and body composition. In Ethiopia, Temesgen et al. showed that women from high‐income families were 75% less likely to be underweight (Temesgen et al., 2015). A cross‐sectional study from Brazil showed that homemaker's women were those presented both low food purchasing capacity and poor nutritional status (low mean BMI) (Silva et al., 2017). This may be important for mothers to have their own money so that they can buy a variety of food, especially ASFs, thereby improving diet diversity and nutrition outcomes.

In this study, results showed about 90% of mothers who receive antenatal had better nutritional status (BMI and body composition). About 90% of women used antenatal care service, showing that Senegalese health's programme is considered to be on a good track to improve neonatal outcomes. Haileslassie et al. showed that women who attended less than three antenatal care visits a year were 4.1 more likely to be underweight (Haileslassie et al., 2013); similarly, Gebre et al. showed that women who had not attended antenatal care were 83% more likely to be underweight (Gebre et al., 2018).

To our knowledge, this is the first study to assess simultaneously body composition in mother–child pairs. However, our study has some limitations, namely, regarding the small sample size and its lack of representativeness, because most women enrolled were rural and of a low socio‐economic status. Nutritional adequacy of women of lower socio‐economic status should be more investigated to know the other underlying causes of malnutrition. However, despite our small sample size, our results highlight a strong association between maternal and child body composition parameters in early life and underline the need for more prospective better know the mechanism of this relationship in both mothers and children, especially in lower income and less educated populations.

## 5 CONCLUSION

This study shows, for the first time, a strong association between maternal and child body composition parameters in early life. Good dietary diversity associated with a better children nutrition status, while being employed and doing reproductive health care seem to benefit the maternal nutritional status. The relationships highlight the need to improve women poverty and empowerment, health care services and food dietary in order to enhance maternal and child nutritional status.

## CONFLICTS OF INTEREST

The authors declare that they have no conflicts of interest.

## CONTRIBUTIONS

All the authors participated in the study design and the implementation of the research project. AB, PMDDS and NSC collected the data. AB, MD and AD were responsible of the quality control of the data and the statistical analyses. AB, AD, NID and PD wrote the manuscript. PMDDS, NSC and SW reviewed the manuscript. All authors read and approved the final manuscript.

## Data Availability

Data are available if required.

## References

[mcn13174-bib-0001] Abubo, M. J. , Ndenge, G. K. , & Mwabu, D. K. (2008). Determinants of children's nutritional status in Kenya: Evidence from demographic and health surveys. Journal of African Economy, 18, 363–387.

[mcn13174-bib-0002] Addo, O. Y. , Stein, A. D. , Fall, C. H. , Gigante, D. P. , Guntupalli, A. M. , Horta, B. L. , Kuzawa, C. W. , Lee, N. , Norris, S. A. , Prabhakaran, P. , Richter, L. M. , Sachdev, H. S. , Martorell, R. , & Consortium on Health Orientated Research in Transitional Societies (COHORTS) Group . (2013). Maternal height and child growth patterns. Journal of Pediatric, 163, 549–554. 10.1016/j.jpeds.2013.02.002 PMC3711792 23477997

[mcn13174-bib-0003] Agence Nationale de la Statistique et de la Démographie (ANSD) . (2017). Enquête Démographique et de Santé à Indicateurs Multiples EDSV‐MICS, 2016. Pp. 380. Dakar, Sénégal. Rapport EDS‐Continue.

[mcn13174-bib-0004] Alemayehu, M. , Medhanyie, A. A. , Berhanu, K. , Gebremariam, Y. , Hailu, T. , Beyene, S. A. , Ahmed, M. , & Mulugeta, A. (2018). The levels of utilization of reproductive, maternal and neonatal health services among women from pastoralist communities in afar, Ethiopia: across‐sectional survey. Ethiopian Journal of Health Development, 32, 34–42.

[mcn13174-bib-0005] Arimond, M. , & Ruel, M. T. (2004). Dietary diversity is associated with child nutritional status: Evidence from 11 Demographic and Health Surveys. Journal of Nutrition, 134, 2579–2585. 10.1093/jn/134.10.2579 15465751

[mcn13174-bib-0056] Badiane, A. , Sylla, Papa M. D. D. , Diouf, A. , Tall, L. , Mbaye, M. S. , Cisse, N. S. , Dossou, N. I. , Wade, S. , & Donnen, P. (2018). Sensory evaluation and consumer acceptability of orange fleshed sweet potato by lactating women and children <2 years old in Kaffrine, Central groundnut Basin of Sénégal. African Journal of Food Science, 12(11), 288–296.

[mcn13174-bib-0006] Barden, E. M. , Zemel, B. S. , Kawchak, D. A. , Goran, M. I. , Ohene‐Frempong, K. , & Stallings, V. A. (2000). Total and resting energy expenditure in children with sickle cell disease. Journal of Pediatric, 136, 73e9. 10.1016/s0022-3476(00)90053-2 10636978

[mcn13174-bib-0007] Black, R. E. , Victora, C. G. , Walker, S. P. , Bhutta, Z. A. , Christian, P. , de Onis, M. , Ezzati, M. , Grantham‐McGregor, S. , Katz, J. , Martorell, R. , Uauy, R. , & the Maternal and Child Nutrition Study Group . (2013). Maternal and child undernutrition and overweight in low‐income and middle‐income countries. Lancet, 382, 427–451. 23746772 10.1016/S0140-6736(13)60937-X

[mcn13174-bib-0008] Bork, K. , Cames, C. , Barigou, S. , Cournil, A. , & Diallo, A. (2012). A summary index of feeding practices is positively associated with height‐for‐age, but only marginally with linear growth, in rural Senegalese infants and toddlers. Journal of Nutrition, 142(6), 1116–1122. 10.3945/jn.112.157602 22535757

[mcn13174-bib-0009] Butte, N. F. , Wong, W. W. , Hopkinson, J. M. , Smith, E. O. , & Ellis, K. J. (2000). Infant feeding mode affects early growth and body composition. Pediatrics, 106, 1355–1366. 10.1542/peds.106.6.1355 11099589

[mcn13174-bib-0010] Christian, P. , & Smith, E. R. (2018). Adolescent undernutrition: Global burden, physiology, and nutritional risks. Annals of Nutrition and Metabolism, 72(4), 316–328. 10.1159/000488865 29730657

[mcn13174-bib-0011] Correia, L. L. , Silva, A. C. , Campos, J. S. , Andrade, F. M. , Machado, M. M. , Lindsay, A. C. , Leite, A. J. , Rocha, H. A. , & Cunha, A. J. (2014). Prevalence and determinants of child undernutrition and stunting in semiarid region of Brazil. Revista Saúde Pública, 48(1), 19–28. 10.1590/S0034-8910.2014048004828 PMC4206126 24789633

[mcn13174-bib-0012] De Lorenzo, A. , Deurenberg, P. , & Pietrantuono, M. (2003). How fat is obese? Acta Diabetology, 40, S254–S257. 10.1007/s00592-003-0079-x 14618486

[mcn13174-bib-0013] Desalegn, B. B. , Lambert, C. , Riedel, S. , Negese, T. , & Biesalski, H. K. (2018). Ethiopian orthodox fasting and lactating mothers: Longitudinal study on dietary pattern and nutritional status in rural Tigray, Ethiopia. International Journal of Environmental Research and Public Health, 15(8), 1767. 10.3390/ijerph15081767 PMC6121597 30126089

[mcn13174-bib-0014] Diouf, A. , Badiane, A. , Manga, N. M. , Dossou, N. I. , Sow, P. S. , & Wade, S. (2016). Daily consumption of ready‐to‐use peanut‐based therapeutic food increased fat free mass, improved anemic status but has no impact on the zinc status of people living with HIV/AIDS: A randomized controlled trial. BMC Public Health, 16, 1. 26728978 10.1186/s12889-015-2639-8 PMC4700615

[mcn13174-bib-0015] Diouf, A. , Diongue, O. , Nde, M. , Idohou‐Dossou, N. , Thiam, M. , & Wade, S. (2018). Validity of bioelectrical impedance analysis in predicting total body water and adiposity among Senegalese school‐aged children. PLoS One, 13(10), e0204486. 10.1371/journal.pone.0204486 30307965 PMC6181292

[mcn13174-bib-0016] Ejlerskov, K. T. , Christensen, L. B. , Ritz, C. , Jensen, S. M. , Molgaard, C. , & Michaelsen, K. F. (2015). The impact of early growth patterns and infant feeding on body composition at 3 years of age. British Journal of Nutrition, 114, 316–327. 10.1017/S0007114515001427 26131962

[mcn13174-bib-0017] Eriksen, H. L. F. , Kesmodel, U. S. , Underbjerg, M. , Kilburn, T. R. , Bertrand, J. , & Mortensen, E. L. (2013). Predictors of intelligence at the age of 5: Family, pregnancy and birth characteristics, postnatal influences, and postnatal growth. PLoS One, 8, e79200. 10.1371/journal.pone.0079200 24236109 PMC3827334

[mcn13174-bib-0018] Food and Agriculture Organization of the United Nations , & Food and Nutrition Technical Assistance III Project . (2016). Minimum dietary diversity for women: A guide for measurement. Rome: FAO.

[mcn13174-bib-0019] Food and Agriculture Organization , & World Health Organization (FAO/WHO) . (2004). Human vitamin and mineral requirements. Report of a Joint FAO/WHO Expert Consultation. (pp. 1–57). Geneva: FAO/WHO.

[mcn13174-bib-0020] Forbes, G. B. (1987). Human body composition. New York: Springer Verlag. 10.1007/978-1-4612-4654-1

[mcn13174-bib-0021] Gebre, B. , Biadgilign, S. , Taddese, Z. , Legesse, T. , & Letebo, M. (2018). Determinants of malnutrition among pregnant and lactating women under humanitarian setting in Ethiopia. BMC Nutrition, 4(1), 11. 10.1186/s40795-018-0222-2 32153875 PMC7050776

[mcn13174-bib-0022] George, S. M. , Ballard‐Barbash, R. , Manson, J. E. , Reedy, J. , Shikany, J. M. , Subar, A. F. , Tinker, L. F. , Vitolins, M. , & Neuhouser, M. L. (2014). Comparing indices of diet quality with chronic disease mortality risk in postmenopausal women in the Women's Health Initiative Observational Study: Evidence to inform national dietary guidance. American Journal of Epidemiology, 180(6), 616–625. 10.1093/aje/kwu173 25035143 PMC4157698

[mcn13174-bib-0023] Gillis, L. J. , Kennedy, L. C. , Gillis, A. M. , & Bar‐Or, O. (2002). Relationship between juvenile obesity, dietary energy and fat intake and physical activity. International Journal of Obesity and Related Metabolic Disorders, 26, 458e63. 12075571 10.1038/sj.ijo.0801967

[mcn13174-bib-0024] Haileslassie, K. , Mulugeta, A. , & Girma, M. (2013). Feeding practices, nutritional status and associated factors of lactating women in Samre Woreda, South Eastern Zone of Tigray, Ethiopia. Nutrition Journal, 12, 28. 10.1186/1475-2891-12-28 23452646 PMC3599359

[mcn13174-bib-0025] Institute of Medicine (U.S.), Panel on Macronutrients, Institute of Medicine (IOM) . (2005). Standing committee on the scientific evaluation of dietary reference intakes: Dietary reference intakes for energy, carbohydrate, fiber, fat, fatty acids, cholesterol, protein, and amino acids. Washington, D.C: National Academies Press.

[mcn13174-bib-0026] International Atomic Energy Agency . (2010). Human health series no. 12: Introduction to body composition assessment using the deuterium dilution technique with analysis of saliva samples by Fourier transform infrared spectrometry. Vienna: IAEA.

[mcn13174-bib-0027] Jehn, M. , & Brewis, A. (2009). Paradoxical malnutrition in mother‐child pairs: Untangling the phenomenon of over‐ and under‐nutrition in underdeveloped economies. Economics and Human Biology, 7(1), 28–35. 10.1016/j.ehb.2009.01.007 19246260

[mcn13174-bib-0028] Kamal, S. M. M. , Hassan, C. H. , & Alam, G. M. (2015). Dual burden of underweight and overweight among women in Bangladesh: Patterns, prevalence, and sociodemographic correlates. Journal of Health Population and Nutrition, 33(1), 92–105. PMC4438653 25995726

[mcn13174-bib-0029] Krasevec, J. , An, X. , Kumapley, R. , Bégin, F. , & Frongillo, E. A. (2017). Diet quality and risk of stunting among infants and young children in low‐ and middle‐income countries. Maternal & Child Nutrition, 13(S2), e12430. 10.1111/mcn.12430 PMC6865990 29032628

[mcn13174-bib-0030] Lander, R. L. , Lander, A. G. , Houghton, L. , WilliamsII, S. M. , Costa‐RibeiroIII, H. , Barreto, D. L. III , Mattos, A. P. , & GibsonI, R. S. (2012). Factors influencing growth and intestinal parasitic infections in preschoolers attending philanthropic daycare centers in Salvador, Northeast Region of Brazil. Cadernos de Saúde Pública, 28, 2177–2188. 10.1590/S0102-311X2012001100017 23147959

[mcn13174-bib-0031] Lohman, T. G. (1986). Applicability of body composition techniques and constants for children and youth. Exercise and Sport Sciences Reviews, 14, 325–357. 3525188

[mcn13174-bib-0032] Mardani, M. , Abbasnezhad, A. , Ebrahimzadeh, F. , Roosta, S. , Rezapour, M. , & Choghakhori, R. (2020). Assessment of nutritional status and related factors of lactating women in the urban and rural areas of Southwestern Iran: A population‐based cross‐sectional study. International Journal of Community‐Based Nursing and Midwifery, 8(1), 73–83. 10.30476/IJCBNM.2019.73924.0 32039281 PMC6969947

[mcn13174-bib-0033] Marinangeli, C. P. F. , & Jones, P. J. H. (2012). Pulse grain consumption and obesity: Effects on energy expenditure, substrate oxidation, body composition, fat deposition and satiety. British Journal of Nutrition, 108, S46–S51. 10.1017/S0007114512000773 22916815

[mcn13174-bib-0034] Martins, I. S. , Marinho, S. P. , de Oliveira, D. C. , & de Araujo, E. A. (2007). Poverty, malnutrition and obesity: Interrelationships among the nutritional status of members of the same family. Ciência & Saúde Coletiva, 12(6), 1553–1565. 10.1590/S1413-81232007000600017 18813492

[mcn13174-bib-0035] Negash, C. , Whiting, S. J. , Henry, C. J. , Belachew, T. , & Hailemariam, T. G. (2015). Association between maternal and child nutritional status in Hula, Rural Southern Ethiopia: A cross sectional study. PLoS One, 10(11), e0142301. 10.1371/journal.pone.0142301 26588687 PMC4654505

[mcn13174-bib-0036] Organisation Mondiale de la Santé . (1996). Utilisation et interprétation de l'anthropométrie. Rapport d'un comité OMS d'experts. Genève: OMS.

[mcn13174-bib-0037] Perez‐Escamilla, R. , Bermudez, O. , Buccini, G. S. , Kumanyika, S. , Lutter, C. K. , Monsivais, P. , & Victora, C. (2018). Nutrition disparities and the global burden of malnutrition. British Medical Journal, 361, k2252. 29899012 10.1136/bmj.k2252 PMC5996967

[mcn13174-bib-0038] Ruel, M. T. , Alderman, H. , & the Maternal and Child Nutrition Study Group . (2013). Nutrition‐sensitive interventions and programs: How can they help to accelerate progress in improving maternal and child nutrition? Lancet, 382, 536–551. 23746780 10.1016/S0140-6736(13)60843-0

[mcn13174-bib-0039] Ruel, M. T. , & Menon, P. (2002). Child feeding practices are associated with child nutritional status in Latin America: Innovative uses of the Demographic and Health Surveys. Journal of Nutrition, 132, 1180–1187. 10.1093/jn/132.6.1180 12042431

[mcn13174-bib-0040] Silva, D. , Valente, A. , Borgues, A. , Dias, C. , Almeida, F. , Cruz, J. L. , Neves, E. , Afonso, C. , & Guerra, A. (2017). Relationship between the mothers' nutritional status with that of a child population from São Tomé Principe, “Africa”. Revista Brasileira de Saúde Materno Infantil, 17(2), 327–335. 10.1590/1806-93042017000200007

[mcn13174-bib-0041] Silveira, K. , Alves, J. , Ferreira, H. , Sawaya, A. L. , & Florêncio, T. M. M. T. (2010). Association between malnutrition in children living in favelas, maternal nutritional status, and environmental factors. Journal of Pediatric, 86(3), 15–20. 10.2223/JPED.1991 20440445

[mcn13174-bib-0042] Smith, L. C. R. U. , Ndiaye, A. , Haddad, L. , & Martorell, R. (2003). The importance of women's status for child nutrition in developing countries. International Food Policy Research Institute. Food and Nutrition Bulletin, 24(3), 287–288.

[mcn13174-bib-0043] Spurr, G. , & Reina, J. C. (1988). Patterns of daily energy expenditure in normal and marginally undernourished school‐aged Colombian children. European Journal of Clinical Nutrition, 42, 819e34. 3234323

[mcn13174-bib-0044] Tang, M. , & Krebs, N. F. (2014). High protein intake from meat as complementary food increases growth but not adiposity in breastfed infants: A randomized trial. The American Journal of Clinical Nutrition, 100(5), 1322–1328. 10.3945/ajcn.114.088807 25332329 PMC4196483

[mcn13174-bib-0045] Temesgen, D. H. , Dessalegn, W. , Habtamu, F. G. , & Dunkana, N. K. (2015). Nutritional status and associated factors among lactating mothers in Nekemte Referral Hospital and Health Centers, Ethiopia. International Journal of Nutrition and Food Sciences, 4(2), 216–222.

[mcn13174-bib-0046] Ukegbu, P. O. (2014). A study of the nutritional status and dietary intake of lactating women in Umuahia, Nigeria. American Journal Health & Research, 2(1), 20–26.

[mcn13174-bib-0047] Van Itallie, T. B. , Yang, U. M. , Heymsfield, S. B. , Funk, C. , & Boileau, R. A. (1990). Height‐normalized Indices of the body's fat‐free mass and fat mass: Potentially useful indicators of nutritional status. American Journal of Clinical Nutrition, 52, 953–959. 10.1093/ajcn/52.6.953 2239792

[mcn13174-bib-0048] Vir, S. C. (2016). Improving women's nutrition imperative for rapid reduction of childhood stunting in South Asia: Coupling of nutrition specific interventions with nutrition sensitive measures essential. Maternal & Child Nutrition, 12(S1), 72–90. 10.1111/mcn.12255 27187909 PMC5084747

[mcn13174-bib-0049] Wang, Z. M. , Pierson, R. N. , & Heymsfield, S. B. (1992). The five‐level model: A new approach to organizing body composition research. American Journal of Clinical Nutrition, 56, 19–28. 10.1093/ajcn/56.1.19 1609756

[mcn13174-bib-0050] Wells, J. C. K. , & Fewtrell, M. S. (2006). Measuring body composition. Archives of Disease in Childhood, 91, 612–617. 10.1136/adc.2005.085522 16790722 PMC2082845

[mcn13174-bib-0059] Wells, J. C. K. , Hawton, K. , Darch, T. , & Lunn, P. G. (2009). Body composition by 2H dilution in Gambian infants: comparison with UK infants and evaluation of simple prediction methods. British Journal of Nutrition, 102, 1776–1782. 10.1017/S0007114509991255 19682404

[mcn13174-bib-0058] Williams, D. P. , Going, S. B. , Lohman, T. G. , Harsha, D. W. , Srinivasan, S. R. , Webber, L. S. , & Berenson, G. S. (1992). Body fatness and risk for elevated blood pressure, total cholesterol, and serum lipoprotein ratios in children and adolescents. American Journal of Public Health, 82(3), 358–363. 10.2105/ajph.82.3.358 1536350 PMC1694353

[mcn13174-bib-0051] World Health Organization , & UNICEF . (2008). Indicators for assessing infant and young child feeding practices: Part 2: Measurement. Geneva: WHO/UNICEF.

[mcn13174-bib-0052] World Health Organization/Multicentre Growth Reference Study Group . (2006). WHO Child Growth Standards: Length/height‐for‐age, weight‐for‐age, weight‐for‐length, weight‐for‐height and body mass index‐for‐age: Methods and development. Geneva: WHO.

[mcn13174-bib-0053] Wosje, K. S. , Khoury, P. R. , Claytor, R. P. , Copeland, K. A. , Hornung, R. W. , Daniels, S. R. , & Kalkwarf, H. J. (2010). Dietary patterns associated with fat and bone mass in young children. American Journal of Clinical Nutrition, 92, 294–303. 10.3945/ajcn.2009.28925 PMC2904032 20519562

[mcn13174-bib-0054] Zemel, M. B. , & Bruckbauer, A. (2013). Effects of a leucine and pyridoxine‐containing nutraceutical on body weight and composition in obese subjects. Diabetes, Metabolic Syndrome and Obesity: Targets and Therapy, 6, 309–315. 10.2147/DMSO.S49623 PMC3755702 24003309

